# Poribohon-BD: Bangladeshi local vehicle image dataset with annotation for classification

**DOI:** 10.1016/j.dib.2020.106465

**Published:** 2020-10-27

**Authors:** Shaira Tabassum, Sabbir Ullah, Nakib Hossain Al-nur, Swakkhar Shatabda

**Affiliations:** Department of Computer Science and Engineering, United International University, Bangladesh

**Keywords:** Vehicle image dataset, Image annotation, Data augmentation, Vehicle classification, Convolutional neural network, Computer vision

## Abstract

Vehicle Classification has become tremendously important due to various applications such as traffic video surveillance, accident avoidance, traffic congestion prevention, bringing intelligent transportation systems. This article presents ‘Poribohon-BD’ dataset for vehicle classification purposes in Bangladesh. The vehicle images are collected from two sources: i) smartphone camera, ii) social media. The dataset contains 9058 labeled and annotated images of 15 native Bangladeshi vehicles such as bus, motorbike, three-wheeler rickshaw, truck, wheelbarrow. Data augmentation techniques have been applied to keep the number of images comparable to each type of vehicle. For labeling the images, LabelImg tool by Tzuta Lin has been used. Human faces have also been blurred to maintain privacy and confidentiality. The dataset is compatible with various CNN architectures such as YOLO, VGG-16, R-CNN, DPM. It is available for research purposes at https://data.mendeley.com/datasets/pwyyg8zmk5/2.

**Specifications Table**

**Table:** 

Subject	Computer Vision and Pattern Recognition
Specific subject area	Vehicle Classification
Type of data	2D-RGB image (JPG) XML file
How data were acquired	Bangladeshi vehicle images are collected from two sources: 1 Smartphone camera 2 Social media (Facebook)
Data format	Raw digital images (.jpg) Image annotation values (.xml)
Parameters for data collection	These images have been captured in following circumstances: 1 Variance in view, pose, and angle of the vehicles. 2 Different lighting conditions such as sunny morning, low light environment, dark in the night. 3 Various weather conditions such as daylight, rainy days.
Description of data collection	The images are collected from roads and highways of Bangladesh using smartphone cameras. 1791 images are generated through data augmentation techniques. Around 4000 images are collected from Facebook.
Data source location	Bangladesh
Data accessibility	Repository name: Poribohon-BD Data identification number: 10.17632/pwyyg8zmk5.2 Direct URL to data: https://data.mendeley.com/datasets/pwyyg8zmk5/2

**Value of the Data**

• This dataset can be used to train deep learning models for vehicle detection, classification, and segmentation purposes.
• Deep learning researchers interested in the area of vehicle identification, segmentation can be benefited using this dataset. More specifically, this dataset will benefit researchers in developing any traffic management applications for Bangladesh.
• The dataset contains 9058 images of 15 Bangladeshi vehicles. It can be extended by increasing the number of images per class and adding some more types of vehicles. The extension of this dataset will improve and increase classification accuracy of deep learning models [1].
• This dataset can be used in multitudes of applications such as identifying unauthorized vehicles, detecting unfit vehicles, reducing exceeding speed, collecting highway toll, counting vehicles, receiving traffic information, checking empty spots in garages.
• Identifying surrounding vehicles is important for a self-driving vehicle [2]. This dataset has no applicable limit in order to bring autonomous vehicle systems in Bangladesh. The advanced applications using this dataset might help the traffic police maintain traffic laws and make a more efficient traffic system.

## 1 Data Description

**Fig. 1:**
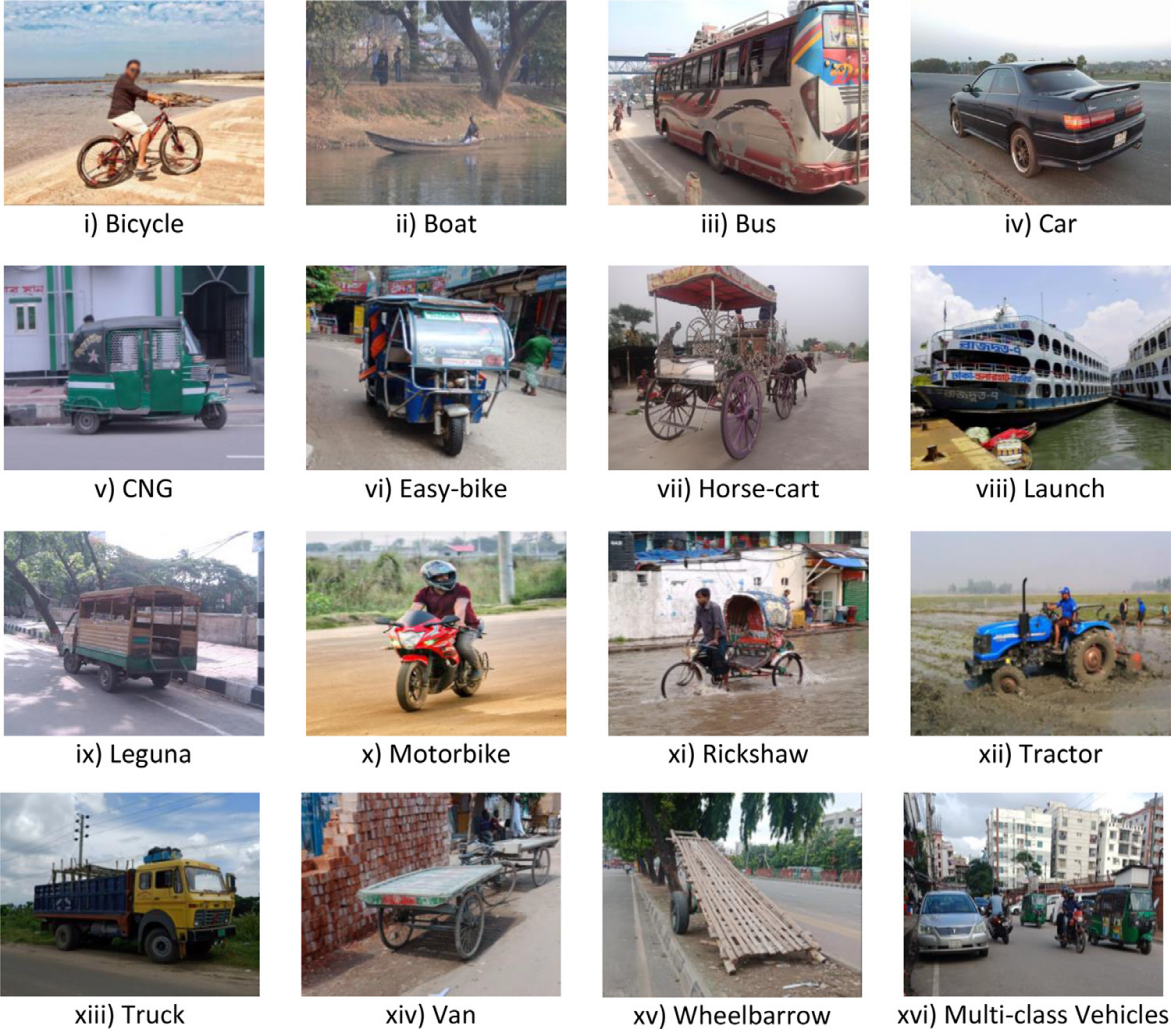
Sample Images of ‘Poribohon-BD’ dataset.
2) Image Annotation Files: The dataset also contains 9058 image annotation files. These files state the exact positions of the objects with labels in the corresponding image. The annotation has been performed manually and the annotated values are stored in XML files. A sample annotation file with corresponding image is given in Fig. 2.

Poribohon-BD is an image dataset of 15 native vehicles of Bangladesh. The vehicles are: i) Bicycle, ii) Boat, iii) Bus, iv) Car, v) CNG, vi) Easy-bike, vii) Horse-cart, viii) Launch, ix) Leguna, x) Motorbike, xi) Rickshaw, xii) Tractor, xiii) Truck, xiv) Van, xv) Wheelbarrow. There are two types of data files in the dataset as follows:

1) Raw Digital Images: The dataset contains a total of 9058 images with a high diversity of poses, angles, lighting conditions, weather conditions, backgrounds. All of the images are in JPG format in the dataset. Some sample images of the dataset are presented in Fig. 1.

**Fig. 2:**
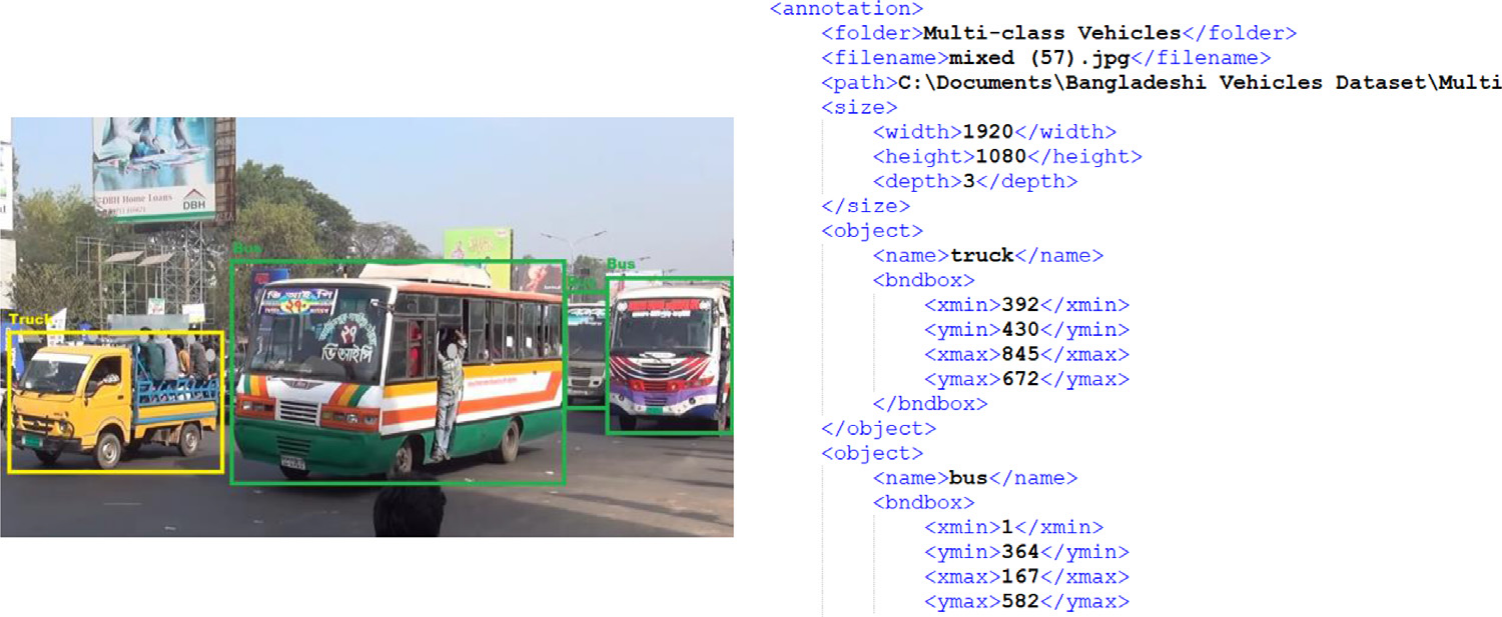
Sample image with manual image annotation file.

The data files are divided into 16 folders. Each folder contains images and annotation files of one single vehicle. The ‘Multi-class Vehicles’ folder contains images and annotation files of multiple types of vehicles. The number of images per class with other details is given in Table 2.

There are multitudes of available datasets to train deep learning models such as COCO, ImageNet, MNIST, CIFAR10, PASCAL VOC. For vehicle detection and classification in developed countries, researchers have released several datasets such as KITTI dataset [3], Waymo dataset [4], Cityscapes dataset [5], ApolloScape dataset [6]. A simple comparison of these public vehicle datasets with Poribohon-BD is given in Table 1.

**Table 1:** A comparison among different public vehicle datasets.

Specifications	KITTY	Waymo	Cityscapes	ApolloScape	Poribohon-BD
Number of images	7481	Around 12 million	25000	701	9058
Annotation	3D bounding boxes	LiDAR box annotations, camera box annotations	Fine annotations, coarse annotations	Semantic annotation	2D bounding boxes
Number of classes	8	4	30	32	15
Number of vehicle classes	5	2	6	6	15
Vehicle related classes	Car, van, truck, cyclist, tram	Vehicles, cyclist	Car, truck, bus, motorcycle, bicycle, caravan	Car, motorcycle, bicycle, truck, bus, tricycle	Bicycle, boat, bus, car, CNG, easy-bike, horse-cart, launch, leguna, motorbike, rickshaw, tractor, truck, van, wheelbarrow
Unique vehicle classes	Tram	-	Caravan	Tricycle	Boat, CNG, easy-bike, horse-cart, launch, leguna, rickshaw, tractor, wheelbarrow

**Table 2:** Data description of ‘Poribohon-BD’ dataset.

Classes	Smartphone Cameras	Internet	Data Augmentation	# Image Files	# Annotation Files	Total Appearance
Bicycle	247	460	-	707	707	1617
Boat	33	580	-	613	613	1974
Bus	112	340	-	452	452	3711
Car	148	560	-	708	708	1698
CNG	202	70	-	533	533	3214
Easy-bike	240	70	261	616	616	2062
Horse-cart	38	90	306	256	256	1581
Launch	-	662	128	662	662	332
Leguna	101	10	-	218	218	1686
Motorbike	124	740	107	864	864	746
Rickshaw	435	60	-	495	495	3386
Tractor	2	215	216	433	433	509
Truck	294	80	362	736	736	1673
Van	307	10	298	615	615	2057
Wheelbarrow	124	-	113	237	237	605
Multi Class	863	50	-	913	913	-
TOTAL:	3270	3997	1791	9058	9058	26851

## 2 Experimental Design, Materials and Methods

The dataset preparation consists of four steps: data collection, data preprocessing, data augmentation and data annotation. This section briefly describes each of these steps to prepare Poribohon-BD dataset.

### 2.1 Data collection

To develop any traffic management application for developing countries like Bangladesh, researchers will need a vast amount of images of different native vehicles. Thus, presenting Poribohon-BD dataset in this article aims to provide such a collection. The images are collected from two different sources:

1) Smartphone Cameras: The images of this dataset have been captured using smartphone cameras from different locations, roads, highways, beaches of Bangladesh. Both images and videos were captured using smartphone cameras. Selected frames from the video files are then converted in still images. Different views, backgrounds, weather conditions, scenarios have been considered while taking the pictures to increase variance in the data.
2) Social Media: Around 4000 images are collected from social media (facebook). The images are taken from different facebook profiles with personal consents. Moreover, privacy issues are resolved by hazing the faces and any personal information.

### 2.2 Data pre-processing

After the data collection phase, all of the images have been converted in JPG format. Due to maintaining privacy and confidentiality, human faces or any other kind of personal information have been blurred in the images.

### 2.3 Data augmentation

Data Augmentation is a popular process in machine learning for increasing the amount and diversity of data. It is a popular solution to reduce overfitting in small datasets [7]. In Poribohon-BD dataset, few data augmentation techniques such as flipping, cropping, color space transformation have been applied to generate 1791 new images. The augmented images are also in JPG format.

### 2.4 Data annotation

An annotation file represents the location of an object in an image by containing the coordinates and label of that object [8]. In this last phase, popular annotation tool LabelImg by Tzuta Lin has been used to cautiously label the images. First of all, each image is opened in this tool one by one. Then, a rectangular shape has been drawn manually to the boundary of an object to specify its exact location in that image by X-Y coordinates. Finally, a label has been assigned such as bus, truck, bicycle to each object. In LabelImg, annotated values are saved as XML files in PASCAL VOC format [9].

## CRediT Author Statement

**Shaira Tabassum:** Methodology, Software, Formal analysis, Data curation, Writing - original draft. **Sabbir Ullah:** Investigation, Data curation, Visualization. **Nakib Hossain Al-nur:** Investigation, Data curation, Visualization. **Swakkhar Shatabda:** Conceptualization, Validation, Writing - original draft, Writing - review & editing, Supervision, Project administration.

## Ethics Statement

The reuse of images from Facebook complies to the terms of use. All the images were acquired with the consent of the people, groups or organizations.

## Declaration of Competing Interest

The authors declare that they have no known competing financial interests or personal relationships which have, or could be perceived to have, influenced the work reported in this article.
